# High variability in the attractiveness of municipally-planted decorative plants to insects

**DOI:** 10.7717/peerj.17762

**Published:** 2024-11-06

**Authors:** Tomer J. Czaczkes, Carsten Breuss, Christoph Kurze

**Affiliations:** Universität Regensburg, Regensburg, Germany

**Keywords:** Urbanisation, Pollinators, Evidence-based policy, Decorative plants, Urban biodiversity

## Abstract

Insect populations are declining globally. A major driver of this decline is land use change, including urbanisation. However, urban environments can also offer a wide range of floral resources to pollinators, through ornamental plantings, but these can vary widely in their attractiveness to insects. Often, the largest single planter of ornamental plants in an urban area is the municipality. Here we evaluated the decorative plantings carried out by the city municipality of Regensburg, Germany, by systematically surveying insect visitations on different plant types in late summer, when forage is often limited for pollinators. We found a 130-fold difference from the least to the most attractive plants, and high variation in which insect groups were attracted to which plants. While honey bees, which are not a conservation concern, were the most common insect visitors, some decorative plants attracted a very large proportion of wild bees, flies, and wasps. Our results demonstrate that there is great scope for increasing the supply of urban forage to pollinators in general, and specific groups in particular, without requiring new decorative plant types to be sourced or planted. We argue that providing local evidence-based guidance to municipalities offers a quick and potentially cost-neutral method for supporting urban insect populations.

## Introduction

Insect populations are declining globally (Blüthgen et al., 2023; Goulson, 2019; Wagner et al., 2021). Of special interest is insect pollinator decline, as pollination is a key ecosystem service offered overwhelmingly by insects (Nath, Singh & Mukherjee, 2023; Potts et al., 2010). Declining pollinator numbers and diversity are set to have large adverse effects on crop production, as well as threatening wild plant species which rely on insect pollination (Bauer & Sue Wing, 2016; Bauer & Wing, 2010; Pérez-Méndez et al., 2020). The reasons for insect pollinator decline are diverse (Potts et al., 2010), and include habitat loss due to land use changes (Ganuza et al., 2022; Peters, Keller & Leonhardt, 2022), pesticide impacts (Brittain et al., 2010), pathogen spillover from managed bees (Manley, Boots & Wilfert, 2015), and ecosystem changes caused by climate change (Vasiliev & Greenwood, 2021), with habitat loss and degradation being perhaps the most important (Ganuza et al., 2022).

The loss and degradation of habitats, and subsequent impact on pollinators, is driven to a large extent by urban development and agricultural intensification (Bates et al., 2011; McKinney, 2006; Winfree et al., 2009). This results, amongst other challenges, in a loss of diverse floral resources (Baude et al., 2016; Fuller, 1987; Jones et al., 2021; Kennedy et al., 2013; Potts et al., 2010). However, recent trends in land use changes may be having a positive effect on resource provision for pollinators; specifically, changes from intensive arable land use to low density urban landscapes. Urban landscapes can be a suitable habitat for pollinators since they often offer a high diversity of ornamental flowering plants (Baldock et al., 2019, 2015; Hall et al., 2017; Honchar & Gnatiuk, 2020). Indeed, such ornamental flowering plants may be planted or bred to offer long periods of flower availability, well into seasons of low resource availability (Couvillon, Schürch & Ratnieks, 2014; Ohlinger et al., 2022; Salisbury et al., 2015). The important role of urban environments in supporting pollinators on heavily impacted landscapes is demonstrated by reports of a positive correlation between urbanisation and biodiversity, to a point (Biella et al., 2022; Garbuzov, Samuelson & Ratnieks, 2015; Lowenstein et al., 2014; Samuelson, Schürch & Leadbeater, 2022; Theodorou et al., 2017). Ornamental plants may fill gaps in the nectar and pollen resources offered by wild plants (Erickson et al., 2020; Salisbury et al., 2015; Seitz, vanEngelsdorp & Leonhardt, 2020). Indeed, cities often support a similar, or even larger, diversity of pollinators compared to agricultural landscapes (Baldock et al., 2015; Hall et al., 2017; Wenzel et al., 2020).

Nonetheless, it is important to note that extensive urbanisation also poses a serious threat to pollinators as well. Highly urbanised areas may be very poor supporters of pollinator communities (Fauviau et al., 2022; Geslin et al., 2016, 2013; Liang et al., 2023). Moreover, not all insect groups respond equally to urbanisation, with some groups, such as below-ground nesting and solitary hymenoptera, being especially negatively impacted by urbanisation (Liang et al., 2023). Only a subset of species from natural areas can adapt or survive in urban areas, with urbanisation thus acting as a filter based on the specific functional traits of the species (Fauviau et al., 2022; Hamblin et al., 2017; Liang et al., 2023; Weber et al., 2023). While low density urbanisation can support a much larger and more diverse pollinator population than intensive agricultural landscapes, this will depend strongly on the land use within the urban area, and especially in the amount of ornamental plants offering food and shelter for pollinators.

However, ornamental plants vary widely in their usefulness to pollinators. Some ornamental plant cultivars, such as double flowers (which have an extra set of petals instead of anthers) offer no nectar or pollen, or have inaccessible nectaries (Comba et al., 1999; Corbet et al., 2001; Garbuzov, Samuelson & Ratnieks, 2015; Honchar & Gnatiuk, 2020). There is a huge variation in attractiveness to pollinators between ornamental plants. For example, Garbuzov, Samuelson & Ratnieks (2015) report a 100-fold difference between the most and least attractive ornamental flowers they surveyed. Even amongst ornamental flowers identified as attractive to pollinators, between a 5- and 40-fold difference in attractiveness from most to least attractive was reported (Marquardt et al., 2021a, 2021b; Palmersheim et al., 2022). Indeed, most ornamental plants sold in UK garden centres are not attractive to pollinators (Garbuzov, Alton & Ratnieks, 2017). Additionally, different plants and cultivars are differentially attractive or useful to different insect groups (Erickson, Patch & Grozinger, 2021; Rollings & Goulson, 2019), with many ornamental cultivars being especially popular with generalist insect species not in need of support, such as *Apis meliffera* and *Bombus terrestris* (Honchar & Gnatiuk, 2020; Peters, Keller & Leonhardt, 2022; Rollings & Goulson, 2019). However, the reverse situation also exists–for example, *Anthemis tinctoria* was reported to attract a wide variety of pollinators such as wild bees, flies, and butterflies, but not honey bees or bumble bees (Rollings & Goulson, 2019). While various floral traits have been found to correlate with attractiveness to pollinators (such as floral area cover (Grindeland, Sletvold & Ims, 2005; Kalaman et al., 2022; Marquardt et al., 2021a), colour (Chapman et al., 2023; Reverté et al., 2016), shape (Chapman et al., 2023; Erickson et al., 2022; Howard et al., 2019), and nutritional offering (Chapman et al., 2023; Comba et al., 1999; Corbet et al., 2001), the huge variety of cultivars and interaction with local conditions make accurate prediction of cultivar attractiveness difficult. As such, Rollings & Goulson (2019) recommend that tests for the attractiveness of a wide variety of ornamental cultivars be carried out, ideally at various sites with varying environmental conditions.

Many municipalities manage extensive decorative flower beds. Such flower beds are often replanted multiple times a year, to provide constant blooms. The municipality is thus often the biggest planter of decorative plants in an area. Frequent replanting means that the municipality can be agile in terms of optimising plantings, and also offers a single entity with which to interact. Municipalities are thus a highly attractive partners for researchers and conservation biologists when attempting to provide evidence-based advice for improving food provision for urban pollinators–although decorative plantings in private and common gardens may represent a larger absolute planted area, and thus private citizens are also important partners to target. The first step in such an attempt is to gather local data on ornamental flower visitations by insects in municipally-planted flower beds. Thus, the aim of this study is to provide these data. We surveyed insect presence on a broad variety of ornamental flowers planted by the City of Regensburg (Germany) municipality, both in order to provide more quantitative data on which plants currently being deployed as ornamentals are most attractive to insects, and in order to provide the municipality data on which plant species they should favour in future planting cycles.

## Methods

The majority of this work was previously published as part of a preprint (https://www.biorxiv.org/content/10.1101/2024.01.25.577170v1).

**Figure 1:**
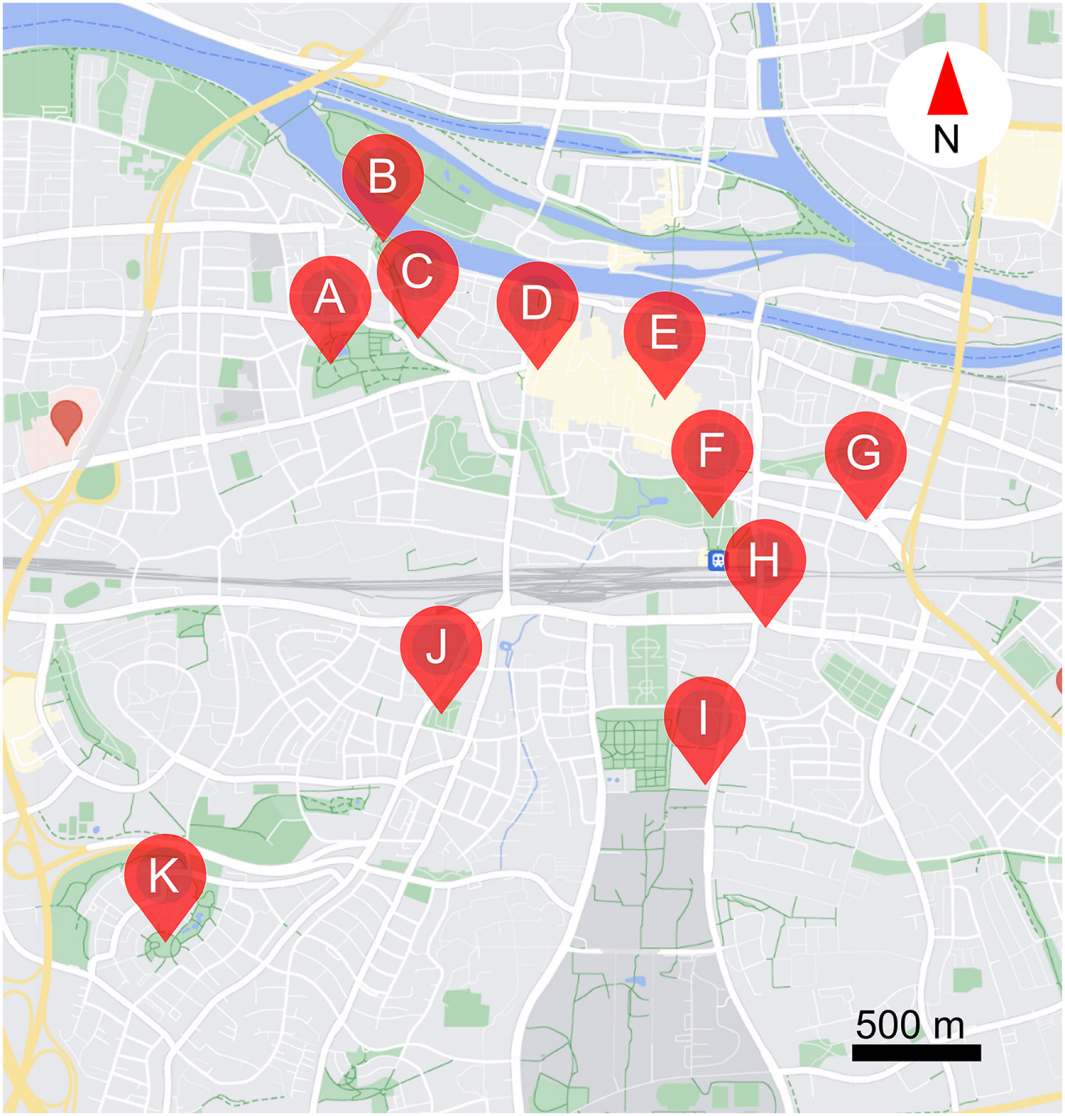
Locations of the 11 study sites within the city of Regensburg. (A) Stadtpark, (B) Herzogpark, (C) Kunstgalerie, (D) Bismarckplatz, (E) Neupfarrplatz, (F) Keplerdenkmal, (G) Stobäusplatz, (H) 24171 Kunstwerk, (I) Ostbayerische Technische Hochschule Regensburg (OTH Regensburg), (J) Ausburger Straße, (K) Kneippbecken. This map was created with GoogleMaps vector data using snazzymaps (https://snazzymaps.com/editor) and Inkscape (https://inkscape.org/).

The study was carried out over 11 public parks and public places in Regensburg, Germany (see Table 1, and Fig. 1). Study locations ranged from the centre to the edge of the city (2.5 km away from city centre). In total, 30 morphogroups (highly similar looking plants) of flowering plants (henceforth plants) were examined, including 25 perennials, one biennial, and four annuals. Four morphogroups contain two different cultivars or species, as they could not be identified before the start of the study (see Table S1). Note that as we could not control what plants were available in each location, the distribution of different plants over the study areas is uneven. The study was carried out from 16/08/2023 to 06/09/2023, when resource availability is low, competition is high, and thus ornamental flowers might be especially important (Couvillon, Schürch & Ratnieks, 2014; Guezen & Forrest, 2021; Ohlinger et al., 2022; Sponsler et al., 2024; Wright et al., 2024). Observations were made between 8:00–11:00 in the morning and 14:00–17:00 in the afternoon, on days when it was not raining or excessively windy. Each site was visited 6 times. Data were only collected from plants in full bloom.

**Table 1:** The study locations with GPS coordinates and a brief description. Map # refers to the labelled map found in Fig. 1.

Map #	Location name	GPS coordinates	Surroundings of location
A	Stadtpark	49°01′13.2″N 12°04′52.8″E	Apartment buildings and other public parks in the surroundings and a busy road nearby
B	Herzogpark	49°01′20.4″N 12°04′56.0"E	Apartment buildings and other public parks in the surroundings
C	Kunstgalerie	49°01′14.5″N 12°04′57.3″E	Apartment buildings and other public parks in the surroundings
D	Bismarckplatz	49°01′14.5″N 12°04′57.3"E	In the city centre, with busy roads and pedestrian zone nearby
E	Neupfarrplatz	49°01′06.5″N 12°05′47.1″E	The centre of the city with a large concrete area nearby
F	Keplerdenkmal	49°00′49.6″N 12°05′58.0″E	Centre of the city with a big public park, very busy roads, a big bus station and a big train station nearby
G	Stobäusplatz	49°00′48.5″N 12°06′29.3″E	At the edge of the city centre, with many apartment buildings and very busy roads nearby
H	24171 Kunstwerk	49°00′35.9″N 12°06′07.0″E	At the edge of the city centre, with many apartment buildings and very busy roads nearby
I	Ostbayerische Technische Hochschule (OTH)	49°00′09.1″N 12°05′55.5″E	Busy road and campus area with extensive green areas nearby
J	Augsburger Straße	49°00′25.6″N 12°05′05.0″E	Many apartment buildings in the surroundings
K	Kneippbecken	48°59′55.0″N 12°04′09.2″E	Edge of the city, with a big public park in proximity, many apartment buildings in the surroundings and arable fields in the distance

In each location, a patch of each plant type available was examined. To do this we carried out a standardised observation following Kalaman et al. (2022) in which a 40 × 60 cm quadrat was placed on the planted area, and all insects visiting flowers of the studied cultivar in the quadrat over 1 min were recorded. We hoped that a slightly longer observation period, rather than the “snapshot” method deployed in other studies (Garbuzov, Samuelson & Ratnieks, 2015; Palmersheim et al., 2022), would allow us to search more closely for smaller or more inconspicuous insects, such as ants or small bees. We assigned recorded insects to one of the following morphotypes: honey bees, wild bees, bumble bees, hover flies, other flies, butterflies, wasps, true bugs, and ants. After the observation we took a digital photograph of the quadrat, and from this calculated the percentage coverage of the plant type being studied. Based on this, we could calculate the insect observations per m^2^ per minute for each observation. We chose to calculate the percentage of specific plant cover of each cultivar in each quadrat, not the percentage of flower cover, as each flower bed has only a limited area for planting, and in this study, we were interested in which plants attract the most visits per unit area of planted flower bed.

Observations were carried out in all areas six times; three times in the morning and three times in the afternoon on different days, leading to at least six observations minimum per plant type. Each observation for each area was carried out on a different day.

The planting (patch) size of each recorded area was noted, although previous studies indicated that the number of insect visitors per unit area is not affected by patch size, at least within the range of patch sizes typically found within gardens (Garbuzov, Samuelson & Ratnieks, 2015).

Statistical analysis was carried out in R (R Core Team, 2023) using the *glmmTMB* package (Magnusson et al., 2020) to perform mixed-effect models. *Post-hoc* pairwise comparisons were carried out using the *emmeans* package (Lenth et al., 2020). In order to compare cultivar attractiveness, we attempted to predict insect visitations per m^2^ by cultivar, with location as a random effect, using a negative binomial distribution family. A similar analysis, carried out only on the subset of wild bee visitations, was used to study variable attractiveness to wild bees. However, in this model a zero-inflation factor was included. To compare differential attractiveness of cultivars to wild- and honeybees, we predicted raw visitation count according to the cultivar and the insect group. This analysis also included a zero inflation factor, but, due to being raw count data, used a Poisson distribution. All models were checked for adequate model fit using the DHARMa package (Hartig, 2020). A complete description of the statistical analysis procedure, including all code and statistical output, is provided in Supplement S2.

## Results

The complete dataset on which this study is based is provided in Supplement S1.

**Figure 2:**
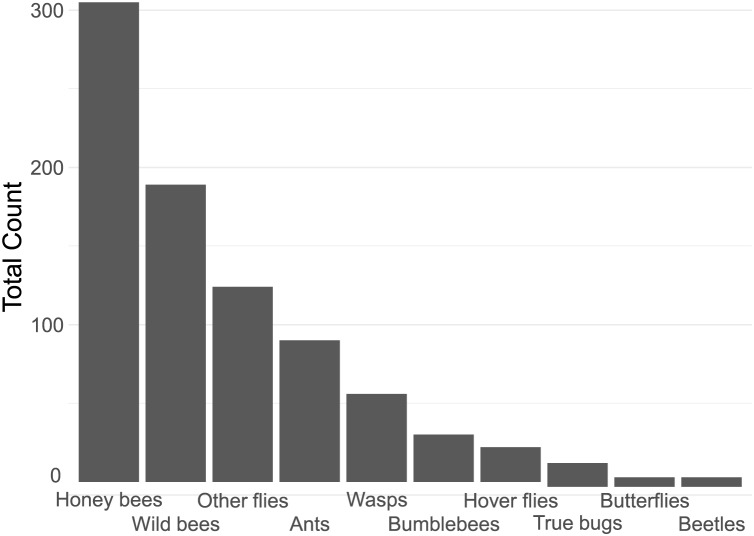
Contribution of each insect group to the total insect count.

In total, we counted 843 insect visitations over 24 different plant types, over a total of c. 80 h of data collection. The four most common insect groups were honey bees, wild bees, flies (excluding hoverflies) and ants (Fig. 2).

**Figure 3:**
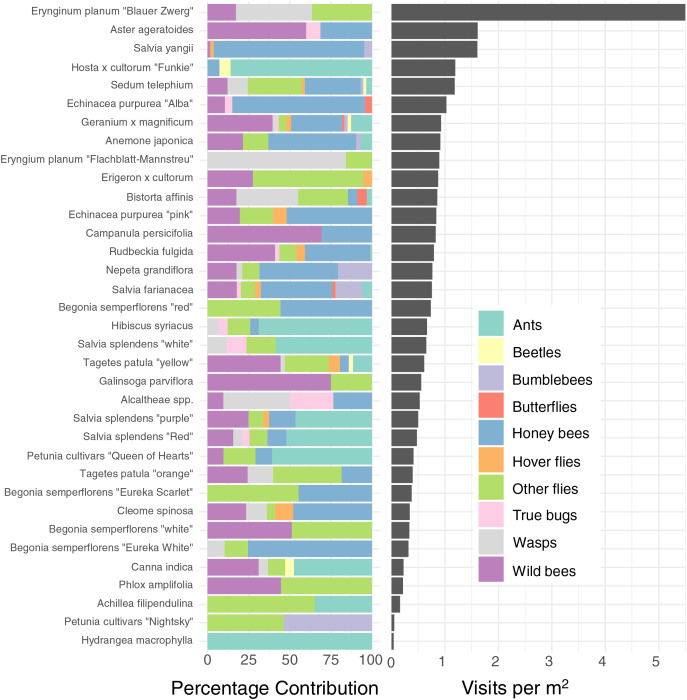
Percentage contribution of each insect type to total visitations for each plant type, and mean visitation rate of all insects to all cultivars, normalised to a rate per m^2^. Left: Percentage contribution of each insect type to total visitations for each plant type. Right: Mean visitation rate of all insects to all cultivars, normalised to a rate per m^2^. Note that some plants, such as Blue Eryngo (*Erynginum planum* “Blaue Zwerg”), were not commonly planted, so may not be a reliable estimate.

The different plants vary very widely in their visitation rate per m^2^ ground cover (glmmTMB, *χ*^2^= 416.29, DF = 34, *P* < 0.0001, Fig. 3, right), with Blue Eryngo (*Erynginum planum* “Blaue Zwerg”) having by far the highest visitation rate (mean 5.5 insects min/m^2^) and French Hydrangea (*Hydrangea* macrophylla) having the lowest (0.041 insects min/m^2^). However, as Blue Eryngo was so rare in the sample (one location, six observations), this measurement is not reliable. The most visited well-sampled plant type was Russian sage (*Salvia yangii*), sampled at three locations with 18 observations, which had a visitation rate of 1.61 insects min/m^2^. As such, taken at face value, there was a 130-fold difference between the most and least visited plant type, and even considering only the more reliable measurements, there is a 40-fold difference between the most and least visited plant types. We refrain from individually comparing pairs of plants for brevity–the entire dataset is provided in Supplement S1.

As well as being differently attractive, different plant types also attracted a different profile of insects (Fig. 3 left). For example, while *Salvia yangii* and *Aster ageratoides* show almost identical overall attractiveness in terms of insect visitations per m^2^ per minute (Fig. 3), *Salvia yangii* is dominated by honey bees, while wild bees are the most common visitors to *Aster ageratoides*. Other plants heavily visited by insects other than honey-and wild bees included the two *Erynginum* cultivars (overwhelmingly wasps and flies), Erigeron × cultorum (mostly flies) and *Alcaltheae spp* (popular with wasps and true bugs).

**Figure 4:**
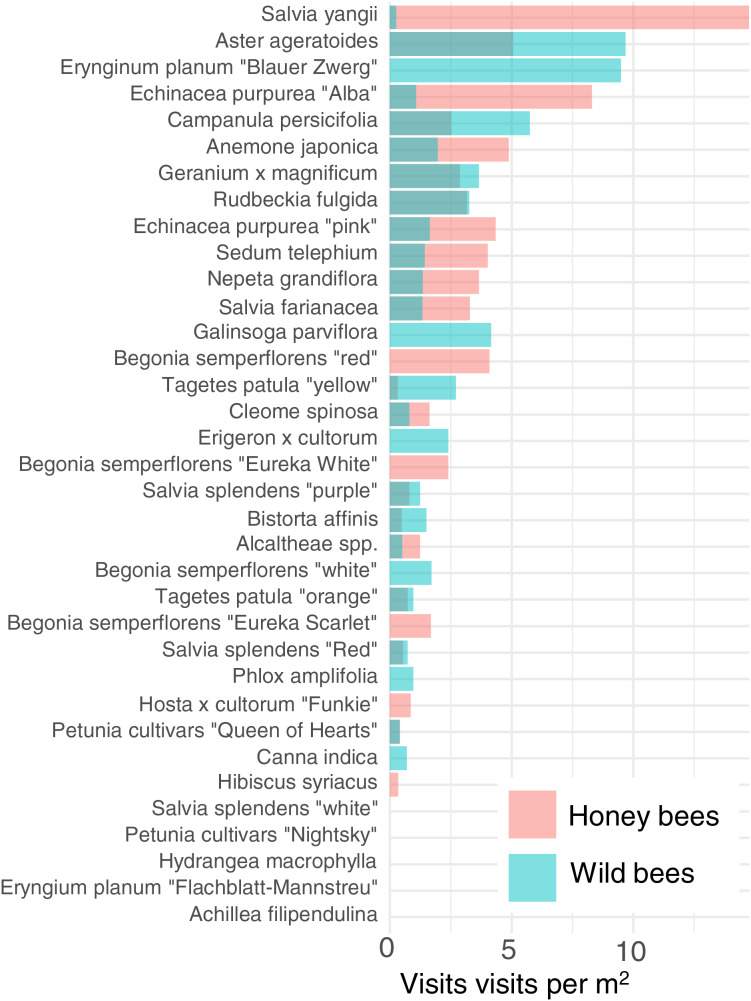
Average visitation rate per M^2^ of different plants by honey bees and wild bees.

In order to reduce dimensionality, we examined the attractiveness of the different cultivars to honey bees and wild bees. Cultivars vary strongly in their attractiveness of honey bees and wild bees (Fig. 4). The cultivar which attracted the most wild bees per m^2^ was *Aster ageratoides* followed closely by *Erynginum planum* “Blaue Zwerg”.

To further reduce dimensionality, and to allow for model convergence, we only consider the top 6 most attractive flowers in terms of mean insect visits per m^2^ for honey bees and wild bees: *Salvia yangii, Aster ageratoides, Erynginum planum* “Blaue Zwerg”, *Echinacea purpurea* “Alba”, *Campanula persicifolia* and *Anemone japonica*. There was a significant interaction between cultivar and insect group (GLMM, *χ*^2^ = 19.5, df = 5, *P* = 0.0015). *Post-hoc* contrast analysis found that more honey bees than wild bees visited *Echinacea purpura “Alba”* (*P* = 0.083) and *Salvia yangii* (*P* = 0.004) while none of the top 6 most attractive cultivars were visited more often by wild bees. However, it is worth noting that no honey bees, but some wild bees, visited the *Erynginum planum* “Blaue Zwerg”, and twice as many wild bees than honey bees visited *Aster ageratoides*. Unfortunately, these plants were too uncommon in the dataset to draw firm conclusions from. We note that these two cultivars were not found in the same location.

## Discussion

We found enormous (up to 40–130 fold) differences of attractiveness between ornamental plants (Fig. 3), mirroring previous studies (Garbuzov, Samuelson & Ratnieks, 2015; Marquardt et al., 2021a, 2021b; Palmersheim et al., 2022). As all plants surveyed were being planted by the municipality already, this suggests that major improvements to the supply of forage for urban pollinators can be affected rapidly with little effort or additional costs.

Notable in their relative absence from our dataset were bumble bees (3.6% of visitations) and butterflies (0.7%, Fig. 2). It is likely that the lack of bumble bees was driven by them not being present: many of the flowers surveyed have previously been reported to be highly attractive to bumblebees, such as *Echinacea purpurea* (Rollings & Goulson, 2019). The absence of bumble bees may well be due to the time of year of the survey–many of the local bumble bee species are coming to the end of their annual life-cycle by late summer. A drought in June and July of that year may have also had an effect. However, the urban parks within Regensburg may also not offer a suitable habitat for them, although higher levels of bumble bee visitations were reported in other surveys of urban pollinators (Honchar & Gnatiuk, 2020; Lowenstein, Matteson & Minor, 2019; Marquardt et al., 2021a). The lack of butterflies may also be explained by the season, although it is likely that the selection of flowers offered were also not especially attractive to honey bees. Plants such as Buddleia or Lantana are especially attractive to butterflies (Shackleton & Ratnieks, 2016). It is also important to note that the survey only occurred in one season, over a 21-day collection range in late summer, from August to early September, while the peak in flower-visiting insect abundance and diversity is earlier, in Spring and early Summer (Guezen & Forrest, 2021; Wright et al., 2024). This likely had a strong effect on the insect visitor mix we report.

Insect groups and species vary widely in their adaptability to urban environments. Beetles, flies, lepidopterans and solitary bees have been previously reported to be highly sensitive to increasing urbanisation, while generalist insects such as *Bombus terrestris* and *Apis melifera* are not (Bergerot et al., 2011; Geslin et al., 2013; Mulieri et al., 2011; Theodorou et al., 2020; Verboven et al., 2014). This only partially overlaps with our findings (see Fig. 2), and indeed other studies, such as Marquardt et al. (2021a) also report a very large proportion of solitary bees on urban flowers, though they also found bumble bees to be rather common. Many wild bees are also generalists, so may be better adapted to coping with a limited or highly variable food supply than specialists, which may be the case in urban environments. Rollings & Goulson (2019) report generally few solitary bees in a plant nursery embedded in a rural environment, except very late in the season (mid-October) when solitary bees suddenly become very common. It is likely that plant type presence, macro-environment, and time of year all strongly affect the number and distribution of attracted insects, highlighting the importance of collecting local data on plant attractiveness.

The majority of the plants surveyed at the time surveyed were attractive to honey and wild bees, but did not attract many visitations from most other groups, especially butterflies and beetles (Fig. 3). At first glance this is disappointing, especially given the presence of locally extremely rare beetles in the city of Regensburg, such as *Nematodes filum* (Regensburg conservation office, personal communication). However, it should be noted again that the survey was carried out well past the peak of flower-visiting insect abundance and diversity. Moreover, it is not clear whether broad conservation of all pollinator groups, or rare species, should be the goal of urban planners. Marquardt et al. (2021a) note that “Pollinator conservation strategies in urban areas differ highly in comparison to the strategies in natural or near-natural environments. In cities, the aim is to increase the overall abundance and diversity of pollinator communities, rather than focusing on rare or endangered species.” An achievable aim may be to support wild bees as a class, while possibly avoiding planting plants which are predominantly attractive to honey bees. Honey bees, as a commercially-bred species, are not a conservation priority, and may indeed compete with other pollinators for resources (although direct measurements of impact are often lacking) (Paini, 2004; Steffan-Dewenter & Tscharntke, 2000; Wojcik et al., 2018), or potentially act as a source of infection (Fürst et al., 2014; Graystock et al., 2016) (but see *e.g*., Dolezal et al. (2016), Mallinger, Gaines-Day & Gratton (2017)). Bumble bee colonies in close proximity to managed honey bee showed longer foraging bout durations (Theodorou et al., 2022). Especially interesting are the decorative thistles, such as the Blue Eryngo, which were very attractive to wild bees, wasps, and flies, but were never visited by honey bees during our observations (Fig. 4). Indeed, examining Fig. 4 highlights several plants, such as *Aster agratoides, Erynginum* (both cultivars studied), *Campanula persicifolia*, and *Galinsoga parviflora* which attract more wild bee than honeybee visits, even though overall we observed 30% more honey bee visits than wild bee visits (Fig. 2). These species, especially those which are also attractive to other animal groups (such as the Erynginum thistles), warrant closer attention as plants to emphasise in municipal plantings. However, it must be noted that the category ‘wild bees’ used is a very broad group, comprising an unknown number of species. More detailed examination of flower visitation, including collection and identification, will be required to fully understand to which species the ‘good for wild bees’ plant species are actually attractive.

A somewhat surprising finding was the relatively common presence of ants on flowers (10.7% of insects, Fig. 2). While surveys of flower attraction to different insect groups are growing in number, ants are very rarely mentioned. It is not clear whether this is because they are ignored, since they rarely act as pollinators (Rostás & Tautz, 2011) and many indeed repel pollinators (Junker, Chung & Blüthgen, 2007), or whether they are not noted. Other smaller insect groups, such as thrips, are also hardly reported. It is possible that the underreporting of small, non- or rarely flying insects is due to the predominance of snapshot surveying, which involves noting what can be seen at a glance. We suspect this will tend to bias samples towards big and mobile groups such as honey bees, bumble bees, and butterflies, and away from more cryptic groups such as small wild bees, ants, and thrips. The approach we took, of surveying a patch for a set period of time, may be less prone to such biases, but also suffers from the issue that some individuals may be counted multiple times. A systematic comparison of both survey types would be a worthwhile endeavour.

It is important to note that the current study, as almost all other such studies, assumes that plants which are more highly visited are more beneficial to pollinators. This may not always be true. Artificially-bred cultivars could act as cognitive traps, providing a super-stimulus of colour or odour without providing a large reward, or only providing an unbalanced reward. While there is a correlation between nutritional offering and attractiveness (Chapman et al., 2023; Comba et al., 1999; Corbet et al., 2001), little information exists about the nectar or pollen productivity of most ornamental plants, nor of the nutritional content of the nectar and pollen offered. Similarly, increased visitations by insects are not always beneficial to the plants (Hargreaves, Harder & Johnson, 2009).

## Conclusions

This study demonstrated that municipally-planted decorative annual flowers vary enormously both in their absolute attractiveness to insects, and in which groups they attract. This suggests that evidence-based changes in municipally-planted flowers could achieve a very rapid improvement in how suitable urban environments are for pollinators, at a very modest financial and organisational expenditure. Indeed, based on the evidence presented in the current study, we have begun a collaboration with the Regensburg municipality to first collect more targeted information about the most promising decorative plants, and ultimately to focus planting on plant species attractive to the local insect groups we wish to promote. However, while promising, some caution is warranted; while this and many studies focus solely on nutritional provision by flowers, not all resources pollinators require come from flowers. Aphids on trees are a very important source of carbohydrates for many insects, and many insects require non-food resources such as resin or nesting sites, which are better provided by trees and bushes (Requier & Leonhardt, 2020). Forage for the larval stages of pollinators is also an important aspect to keep in mind. Improving the attractiveness of municipally-planted flowers is a relatively quick and easy approach, with potentially large rewards, but it can only be one part of a larger programme for supporting urban biodiversity.

## Supplemental Information

10.7717/peerj.17762/supp-1 Supplemental Information 1 Complete raw data including metadata.

10.7717/peerj.17762/supp-2 Supplemental Information 2 The complete analysis code and output.

10.7717/peerj.17762/supp-3 Supplemental Information 3 Complete analysis code and output (PDF).

10.7717/peerj.17762/supp-4 Supplemental Information 4 Plant groups studied. The complete list of plant morphogroups studies, with information about the species or cultivars they contained.

## Additional Information and Declarations

### Data Availability

The following information was supplied regarding data availability: The complete raw data and the code and statistical output are available in the Supplemental Files.
