# Supplementary material for: High variability in the attractiveness of municipally-planted decorative plants to insects
Source: PeerJ. 2024 Nov 6;12:e17762. doi: 10.7717/peerj.17762 (PMC11549908; doi:10.7717/peerj.17762)
Supplement: Supplemental Information 2 [file peerj-12-17762-s002.html]

High variability in the attractiveness of municipally-planted ecorative plants to pollinators


# High variability in the attractiveness of municipally-planted ecorative plants to pollinators

#### Tomer J Czaczkes, Carsten Breuss, and Christoph Kurze

#### 2024-04-26

# packages

```
library (ggplot2)
library(readxl)
library(forcats)
library (glmmTMB)
library (dplyr)
library (DHARMa)
library (multcomp)
library(emmeans)
library (eoffice)
library(gridExtra)
library(car)
```

# input data, subsetting

```
data <- read_excel("municipal_pollinator_data_merged_bumblebees.xlsx", 
    sheet = "global_database_goodnames")

ants <- subset (data, insect_group == "Ants")
flies <- subset (data, insect_group == "Other flies")
honeybees <- subset (data, insect_group == "Honey bees")
wasps <- subset (data, insect_group == "Wasps")
wildbees <- subset (data, insect_group == "Wild bees")

bees_and_wildbees <- rbind(honeybees, wildbees)
bees_wildbees_flies_wasps <- rbind(honeybees, wildbees, flies, wasps)
```

# plots

## plot of all insect visitation rates for all cultivars

First some summary statistics: mean visitation rate for each
cultivar

```
mean_insectsM2 <- data %>%
  group_by(cultivar) %>%
  summarise(mean_insects_m2 = mean(insects_m2_full_coverage, na.rm = TRUE))

mean_insectsM2
```

```
## # A tibble: 35 × 2
##    cultivar                                   mean_insects_m2
##    <chr>                                                <dbl>
##  1 "Achillea filipendulina"                             0.160
##  2 "Alcaltheae spp."                                    0.529
##  3 "Anemone japonica"                                   0.914
##  4 "Aster ageratoides"                                  1.61 
##  5 "Begonia semperflorens \"Eureka Scarlet\""           0.377
##  6 "Begonia semperflorens \"Eureka White\""             0.318
##  7 "Begonia semperflorens \"red\""                      0.735
##  8 "Begonia semperflorens \"white\""                    0.334
##  9 "Bistorta affinis"                                   0.857
## 10 "Campanula persicifolia"                             0.828
## # ℹ 25 more rows
```

now some descriptive plots

```
# mean bar plot
all_insects_by_cultivar_plot <- ggplot(data, aes(x = reorder(cultivar, insects_m2_full_coverage), y = insects_m2_full_coverage)) +
  scale_y_continuous(expand = c(0, 0)) +
  coord_cartesian(ylim = c(0, 6)) +
  stat_summary(fun.y = "mean", geom = "bar", size = 3) +
  theme_minimal() +
  theme(axis.text.x = element_text(angle = 0, hjust = 1, vjust = 0.3),
        axis.text.y = element_text(angle = 0, hjust = 1, vjust = 0.5)) +  # Set angle to 0 for the Y-axis text
  labs(x = "Insects per m2", y = "cultivar")

# Rotate the plot by 90 degrees
all_insects_by_cultivar_plot <- all_insects_by_cultivar_plot + coord_flip()
```

```
## Coordinate system already present. Adding new coordinate system, which will
## replace the existing one.
```

```
plot (all_insects_by_cultivar_plot)
```

```
#topptx (all_insects_by_cultivar_plot, "fig2_all_insects_by_cultivar_plot.pptx", width = 6, height = 8) #this function is used to export the plot to powerpoint for further editiing and then including in the manuscript.Commented out for the markdown file
```

Now individual plots of cultivar attractiveness forthe top 4 most
common insect groups (honeybees wildbees flies ants wasps)

```
insectplot_facet_common <- ggplot (bees_wildbees_flies_wasps, aes(x = reorder(cultivar,insects_m2_full_coverage), y = insects_m2_full_coverage))+
  facet_wrap(~ insect_group, nrow = 4, ncol = 1)+
  scale_y_continuous(expand = c(0, 0)) + # forces X axis to 0, but in this case is overriden by ribbon
  coord_cartesian(ylim = c(0, 26)) +
  stat_summary(fun.y = "mean", geom = "bar",  size = 3, fill = "red") +
  ylab("Average insects per M2") +
  xlab("Cultivar") +
  theme_bw(15) + 
  theme(axis.text.x = element_text(angle = 90, hjust = 1,vjust = 0.3))


plot (insectplot_facet_common)
```

## Total contributions of cultivars and insect groups, in terms of insects per m2

### Contribution of each insect group to total

First some summary statistics

```
data_summary <- data %>%
  group_by(insect_group) %>%
  summarise(total_count = sum(count)) %>%
  mutate(percentage = (total_count / sum(total_count)) * 100) %>%
  arrange(desc(percentage))

data_summary
```

```
## # A tibble: 10 × 3
##    insect_group total_count percentage
##    <chr>              <dbl>      <dbl>
##  1 Honey bees           305     36.2  
##  2 Wild bees            189     22.4  
##  3 Other flies          124     14.7  
##  4 Ants                  90     10.7  
##  5 Wasps                 56      6.64 
##  6 Bumblebees            30      3.56 
##  7 Hover flies           22      2.61 
##  8 True bugs             15      1.78 
##  9 Beetles                6      0.712
## 10 Butterflies            6      0.712
```

first just one bar chart showing contribution of all insect
groups

```
# Calculate the total count for each insect group
total_counts <- data %>%
  group_by(insect_group) %>%
  summarise(total_count = sum(count)) %>%
  arrange(desc(total_count))  # Order by total count in descending order

# Create a bar chart
insect_contribution_plot <- ggplot(total_counts, aes(x = reorder(insect_group, -total_count), y = total_count)) +
  geom_bar(stat = "identity") +
  labs( x = "Insect Group",
       y = "Total Count") +
  theme_minimal() 

plot (insect_contribution_plot)
```

```
#topptx (insect_contribution_plot, "insect_contribution_plot.pptx", width = 6, height = 6)
```

### Insect group visitation proportion by cultivar

This helps us see who the visitors are to each cultivar

```
# Calculate the percentage contribution of 'insects_m2_full_coverage' for each 'insect_group' within each 'cultivar'
data_percent <- data %>%
  group_by(cultivar, insect_group) %>%
  summarise(total_insects_m2 = sum(insects_m2_full_coverage),
            original_values = mean(insects_m2_full_coverage)) %>%
  group_by(cultivar) %>%
  mutate(percentage = total_insects_m2 / sum(total_insects_m2) * 100)
```

```
## `summarise()` has grouped output by 'cultivar'. You can override using the
## `.groups` argument.
```

```
# Create a stacked bar chart showing the percentage contribution
filledstackedplot_cultivar <- ggplot(data_percent, aes(x = reorder(cultivar, original_values), y = percentage, fill = insect_group)) +
  geom_bar(stat = "identity") +
  labs(y = "Cultivar", x = "Percentage Contribution (%)") +  # Swap x and y axis labels
  scale_fill_brewer(palette = "Set3") +
  theme_minimal() +
theme(axis.text.y = element_text(angle = 0, hjust = 1, vjust = 0.3)) +

coord_flip()

# Display the plot
plot(filledstackedplot_cultivar)
```

```
#topptx (filledstackedplot_cultivar, "fig3_filledstackedplot_cultivar.pptx", width = 6, height = 8)
```

## total planted area size vs insect visitation rate

Various publications claim that there is no relationship between
planted area size and insect visitation rates per unit area. I will
quickly confirm visually.

```
ggplot (data, aes(x = total_area_cultivars, y = insects_m2_full_coverage)) +
geom_jitter(width = 10, height = 1, alpha = 0.3) + 
  geom_smooth(method = "lm") + 
  theme_bw(15) + 
  theme(axis.text.x = element_text(angle = 90, hjust = 1)) +
  labs( x = "total area of planted plants in location", y = "Insects per m2")
```

```
## `geom_smooth()` using formula = 'y ~ x'
```

Confirmed. No apparent effect. Naturally, this is also heavily skewed by
the zero inflation, but certainly nothing obvious here. A quick
statistical analysis to be found in the stats part.

## Wild bees and honeybees - plots

To reduce dimensionality and persue clear questions, here we are
focussing in on just wild bees and honeybees. Is there an overlap
between wild bees and honeybees? What plants do wild bees like but
honeybees don’t?

```
fig3_honey_wildbees <- ggplot (bees_and_wildbees, aes(x = reorder(cultivar,insects_m2_full_coverage), y = insects_m2_full_coverage, fill = insect_group))+
  scale_y_continuous(expand = c(0, 0)) + # forces X axis to 0, but in this case is overriden by ribbon
  coord_cartesian(ylim = c(0, 20)) +
  stat_summary(fun.y = "mean", geom = "bar", shape = 23, size = 3, alpha = 0.5) +
  theme_minimal() + 
  theme(axis.text.x = element_text(angle = 90, hjust = 1, vjust = 0.3)) +  # hjust and vjust nudge the x-axis label positions 
  labs( x = "cultivar", y = "Insect visits per m2") +
  coord_flip()
```

```
## Coordinate system already present. Adding new coordinate system, which will
## replace the existing one.
```

```
plot (fig3_honey_wildbees)
```

```
#topptx (fig3_honey_wildbees, "fig3_wildvshoney.pptx", width = 6, height = 6)
```

some descriptive states here:

sum of count

```
# Calculate the sum of "count" for each combination of "cultivar" and "insect_group"
bees_and_wildbees %>%
  group_by(cultivar, insect_group) %>%
  summarise(total_count = sum(count))
```

```
## `summarise()` has grouped output by 'cultivar'. You can override using the
## `.groups` argument.
```

```
## # A tibble: 70 × 3
## # Groups:   cultivar [35]
##    cultivar                                   insect_group total_count
##    <chr>                                      <chr>              <dbl>
##  1 "Achillea filipendulina"                   Honey bees             0
##  2 "Achillea filipendulina"                   Wild bees              0
##  3 "Alcaltheae spp."                          Honey bees             2
##  4 "Alcaltheae spp."                          Wild bees              1
##  5 "Anemone japonica"                         Honey bees            27
##  6 "Anemone japonica"                         Wild bees             12
##  7 "Aster ageratoides"                        Honey bees             7
##  8 "Aster ageratoides"                        Wild bees             13
##  9 "Begonia semperflorens \"Eureka Scarlet\"" Honey bees             4
## 10 "Begonia semperflorens \"Eureka Scarlet\"" Wild bees              0
## # ℹ 60 more rows
```

```
bees_and_wildbees %>%
  group_by(insect_group) %>%
  summarise(total_count = sum(count))
```

```
## # A tibble: 2 × 2
##   insect_group total_count
##   <chr>              <dbl>
## 1 Honey bees           305
## 2 Wild bees            189
```

# statistical analysis

Questions we want to ask: - do cultivars differ in attractiveness
overall? - do cultivears differ in attractiveness for specific target
groups? - does total patch area affect insect visitation rates - then do
pairwise comparisons - which cultivars has more wild bees than
honeybees?

## do cultivars differ in attractiveness?

```
m1 <- glmmTMB(insects_m2_full_coverage ~ cultivar 
              + (1 | location),
              ziformula=~1,
              family=nbinom1,
              data = data)
```

looking at results, testing model fit

```
# testing model fit
simulateResiduals(m1, n = 500, plot = T) #dharma
```

```
## DHARMa:testOutliers with type = binomial may have inflated Type I error rates for integer-valued distributions. To get a more exact result, it is recommended to re-run testOutliers with type = 'bootstrap'. See ?testOutliers for details
```

```
## Object of Class DHARMa with simulated residuals based on 500 simulations with refit = FALSE . See ?DHARMa::simulateResiduals for help. 
##  
## Scaled residual values: 0.7908908 0.966 0.988 0.4696725 0.3231412 0.5229729 0.06865622 0.1879041 0.8175542 0.8212179 0.8565376 0.4296221 0.964 0.1516091 0.01738105 0.6381312 0.2055175 0.4215931 0.4491866 0.2059352 ...
```

```
# top level summary
Anova (m1)
```

```
## Analysis of Deviance Table (Type II Wald chisquare tests)
## 
## Response: insects_m2_full_coverage
##           Chisq Df Pr(>Chisq)    
## cultivar 416.29 34  < 2.2e-16 ***
## ---
## Signif. codes:  0 '***' 0.001 '**' 0.01 '*' 0.05 '.' 0.1 ' ' 1
```

```
#adjust for multiple comparisons
adjX <- glht(m1) # multcomp

summary(adjX, test = adjusted("BH"))
```

```
## 
##   Simultaneous Tests for General Linear Hypotheses
## 
## Fit: glmmTMB(formula = insects_m2_full_coverage ~ cultivar + (1 | 
##     location), data = data, family = nbinom1, ziformula = ~1, 
##     dispformula = ~1)
## 
## Linear Hypotheses:
##                                                     Estimate Std. Error z value
## (Intercept) == 0                                     1.69469    0.60692   2.792
## cultivarAlcaltheae spp. == 0                         0.14181    0.64412   0.220
## cultivarAnemone japonica == 0                        0.59901    0.61460   0.975
## cultivarAster ageratoides == 0                       0.40294    0.62316   0.647
## cultivarBegonia semperflorens "Eureka Scarlet" == 0  0.53668    0.65169   0.824
## cultivarBegonia semperflorens "Eureka White" == 0   -0.04651    0.62876  -0.074
## cultivarBegonia semperflorens "red" == 0             0.23150    0.72878   0.318
## cultivarBegonia semperflorens "white" == 0           0.25822    0.68265   0.378
## cultivarBistorta affinis == 0                        0.37178    0.62214   0.598
## cultivarCampanula persicifolia == 0                  0.84611    0.66669   1.269
## cultivarCanna indica == 0                            0.16360    0.64022   0.256
## cultivarCleome spinosa == 0                         -0.01248    0.62178  -0.020
## cultivarEchinacea purpurea "Alba" == 0               0.45830    0.62413   0.734
## cultivarEchinacea purpurea "pink" == 0               0.50941    0.61761   0.825
## cultivarErigeron x cultorum == 0                     0.75820    0.63069   1.202
## cultivarErynginum planum "Blauer Zwerg" == 0         2.40088    0.61694   3.892
## cultivarEryngium planum "Flachblatt-Mannstreu" == 0  1.59038    0.64522   2.465
## cultivarGalinsoga parviflora == 0                    0.26557    0.73107   0.363
## cultivarGeranium x magnificum == 0                   0.40775    0.60995   0.669
## cultivarHibiscus syriacus == 0                       0.32815    0.63489   0.517
## cultivarHosta x cultorum "Funkie" == 0               0.82405    0.64045   1.287
## cultivarHydrangea macrophylla == 0                  -3.18657    1.22587  -2.599
## cultivarNepeta grandiflora == 0                      0.22392    0.62556   0.358
## cultivarPetunia cultivars "Nightsky" == 0           -3.23855    0.97183  -3.332
## cultivarPetunia cultivars "Queen of Hearts" == 0     0.46119    0.66103   0.698
## cultivarPhlox amplifolia == 0                        0.56841    0.69933   0.813
## cultivarRudbeckia fulgida == 0                       0.39484    0.60951   0.648
## cultivarSalvia farianacea == 0                       0.31851    0.61009   0.522
## cultivarSalvia splendens "purple" == 0               0.70736    0.62108   1.139
## cultivarSalvia splendens "Red" == 0                  0.45030    0.61740   0.729
## cultivarSalvia splendens "white" == 0                0.33364    0.65074   0.513
## cultivarSalvia yangii == 0                           1.00350    0.61436   1.633
## cultivarSedum telephium == 0                         0.43070    0.61380   0.702
## cultivarTagetes patula "orange" == 0                 0.53862    0.63136   0.853
## cultivarTagetes patula "yellow" == 0                 0.46713    0.62104   0.752
##                                                     Pr(>|z|)   
## (Intercept) == 0                                     0.06106 . 
## cultivarAlcaltheae spp. == 0                         0.87580   
## cultivarAnemone japonica == 0                        0.78809   
## cultivarAster ageratoides == 0                       0.78809   
## cultivarBegonia semperflorens "Eureka Scarlet" == 0  0.78809   
## cultivarBegonia semperflorens "Eureka White" == 0    0.96872   
## cultivarBegonia semperflorens "red" == 0             0.84762   
## cultivarBegonia semperflorens "white" == 0           0.84044   
## cultivarBistorta affinis == 0                        0.78835   
## cultivarCampanula persicifolia == 0                  0.78809   
## cultivarCanna indica == 0                            0.87314   
## cultivarCleome spinosa == 0                          0.98398   
## cultivarEchinacea purpurea "Alba" == 0               0.78809   
## cultivarEchinacea purpurea "pink" == 0               0.78809   
## cultivarErigeron x cultorum == 0                     0.78809   
## cultivarErynginum planum "Blauer Zwerg" == 0         0.00349 **
## cultivarEryngium planum "Flachblatt-Mannstreu" == 0  0.09595 . 
## cultivarGalinsoga parviflora == 0                    0.84044   
## cultivarGeranium x magnificum == 0                   0.78809   
## cultivarHibiscus syriacus == 0                       0.78835   
## cultivarHosta x cultorum "Funkie" == 0               0.78809   
## cultivarHydrangea macrophylla == 0                   0.08171 . 
## cultivarNepeta grandiflora == 0                      0.84044   
## cultivarPetunia cultivars "Nightsky" == 0            0.01507 * 
## cultivarPetunia cultivars "Queen of Hearts" == 0     0.78809   
## cultivarPhlox amplifolia == 0                        0.78809   
## cultivarRudbeckia fulgida == 0                       0.78809   
## cultivarSalvia farianacea == 0                       0.78835   
## cultivarSalvia splendens "purple" == 0               0.78809   
## cultivarSalvia splendens "Red" == 0                  0.78809   
## cultivarSalvia splendens "white" == 0                0.78835   
## cultivarSalvia yangii == 0                           0.59723   
## cultivarSedum telephium == 0                         0.78809   
## cultivarTagetes patula "orange" == 0                 0.78809   
## cultivarTagetes patula "yellow" == 0                 0.78809   
## ---
## Signif. codes:  0 '***' 0.001 '**' 0.01 '*' 0.05 '.' 0.1 ' ' 1
## (Adjusted p values reported -- BH method)
```

```
#emmeans

meanie <- emmeans(m1, pairwise ~   cultivar) 
# print (meanie) # no point plotting all pairwise comparisons for all pairs - we'd have a heck of a lot of pairs. Just look at the plot.
plot (meanie)
```

## honey vs wild bees - pairwise comps

Going to use the raw count, because we’re going to compare only
within cultivar. Means we can use poisson distribution too

Going to only look at top 6 most attractive cultivars:

unique(data$cultivar)

```
# List of cultivars to filter
selected_cultivars <- c("Salvia yangii", "Aster ageratoides", "Erynginum planum \"Blauer Zwerg\"", "Echinacea purpurea \"Alba\"", "Campanula persicifolia", "Anemone japonica")

# Creating a subset dataset containing only the specified cultivars
top6cultivars <- bees_and_wildbees[bees_and_wildbees$cultivar %in% selected_cultivars, ]
```

make a figure, check all is in order

```
ggplot (top6cultivars , aes(x = cultivar, y = count, fill = insect_group))+
  scale_y_continuous(expand = c(0, 0)) + # forces X axis to 0, but in this case is overriden by ribbon
  coord_cartesian(ylim = c(0, 45)) +
  stat_summary(fun.y = "sum", geom = "bar", shape = 23, size = 3, alpha = 0.5) +
  theme_bw(12) + 
  theme(axis.text.x = element_text(angle = 90, hjust = 1, vjust = 0.3)) +  # hjust and vjust nudge the x-axis label positions +
  labs( x = "cultivar", y = "Total insects counted", title ="Sum count honey and wildbees, top 6 cutlivars")
```

```
## Warning in stat_summary(fun.y = "sum", geom = "bar", shape = 23, size = 3, :
## Ignoring unknown parameters: `shape`
```

ok now lets see if we get do sensible modelling and pairwise
comparisons with this

```
##NOTE IM USING THE RAW COUNT HERE, NOT THE M2 VARIABLE.

#model
m2beestop6 <- glmmTMB(count ~ as.factor(cultivar) * insect_group
              + (1 | location),
              ziformula=~1,
              family=poisson,
              data = top6cultivars)

Anova (m2beestop6)
```

```
## Analysis of Deviance Table (Type II Wald chisquare tests)
## 
## Response: count
##                                    Chisq Df Pr(>Chisq)   
## as.factor(cultivar)               9.0092  5   0.108698   
## insect_group                      6.8307  1   0.008960 **
## as.factor(cultivar):insect_group 19.5148  5   0.001541 **
## ---
## Signif. codes:  0 '***' 0.001 '**' 0.01 '*' 0.05 '.' 0.1 ' ' 1
```

```
#Dharma
simulateResiduals(m2beestop6, n = 500, plot = T) #dharma
```

```
## Object of Class DHARMa with simulated residuals based on 500 simulations with refit = FALSE . See ?DHARMa::simulateResiduals for help. 
##  
## Scaled residual values: 0.7831745 0.9960342 0.3899917 0.8681415 0.3261663 0.8148226 0.3691393 0.4257348 0.949657 0.1081472 0.4299255 0.954141 0.9653428 0.9459946 0.7635606 0.8870011 0.2395449 0.5948557 0.04640933 0.8098306 ...
```

```
adjX <- glht(m2beestop6) # multcomp
summary(adjX, test = adjusted("BH"))
```

```
## 
##   Simultaneous Tests for General Linear Hypotheses
## 
## Fit: glmmTMB(formula = count ~ as.factor(cultivar) * insect_group + 
##     (1 | location), data = top6cultivars, family = poisson, ziformula = ~1, 
##     dispformula = ~1)
## 
## Linear Hypotheses:
##                                                                                 Estimate
## (Intercept) == 0                                                              -9.271e-02
## as.factor(cultivar)Aster ageratoides == 0                                      8.551e-03
## as.factor(cultivar)Campanula persicifolia == 0                                -1.264e+00
## as.factor(cultivar)Echinacea purpurea "Alba" == 0                              5.316e-01
## as.factor(cultivar)Erynginum planum "Blauer Zwerg" == 0                       -1.957e+01
## as.factor(cultivar)Salvia yangii == 0                                          9.413e-01
## insect_groupWild bees == 0                                                    -7.861e-01
## as.factor(cultivar)Aster ageratoides:insect_groupWild bees == 0                1.389e+00
## as.factor(cultivar)Campanula persicifolia:insect_groupWild bees == 0           1.451e+00
## as.factor(cultivar)Echinacea purpurea "Alba":insect_groupWild bees == 0       -1.266e+00
## as.factor(cultivar)Erynginum planum "Blauer Zwerg":insect_groupWild bees == 0  1.992e+01
## as.factor(cultivar)Salvia yangii:insect_groupWild bees == 0                   -2.897e+00
##                                                                               Std. Error
## (Intercept) == 0                                                               2.922e-01
## as.factor(cultivar)Aster ageratoides == 0                                      5.530e-01
## as.factor(cultivar)Campanula persicifolia == 0                                 1.038e+00
## as.factor(cultivar)Echinacea purpurea "Alba" == 0                              3.520e-01
## as.factor(cultivar)Erynginum planum "Blauer Zwerg" == 0                        8.003e+03
## as.factor(cultivar)Salvia yangii == 0                                          3.252e-01
## insect_groupWild bees == 0                                                     3.624e-01
## as.factor(cultivar)Aster ageratoides:insect_groupWild bees == 0                6.188e-01
## as.factor(cultivar)Campanula persicifolia:insect_groupWild bees == 0           1.300e+00
## as.factor(cultivar)Echinacea purpurea "Alba":insect_groupWild bees == 0        8.461e-01
## as.factor(cultivar)Erynginum planum "Blauer Zwerg":insect_groupWild bees == 0  8.003e+03
## as.factor(cultivar)Salvia yangii:insect_groupWild bees == 0                    1.080e+00
##                                                                               z value
## (Intercept) == 0                                                               -0.317
## as.factor(cultivar)Aster ageratoides == 0                                       0.015
## as.factor(cultivar)Campanula persicifolia == 0                                 -1.217
## as.factor(cultivar)Echinacea purpurea "Alba" == 0                               1.510
## as.factor(cultivar)Erynginum planum "Blauer Zwerg" == 0                        -0.002
## as.factor(cultivar)Salvia yangii == 0                                           2.894
## insect_groupWild bees == 0                                                     -2.169
## as.factor(cultivar)Aster ageratoides:insect_groupWild bees == 0                 2.244
## as.factor(cultivar)Campanula persicifolia:insect_groupWild bees == 0            1.116
## as.factor(cultivar)Echinacea purpurea "Alba":insect_groupWild bees == 0        -1.496
## as.factor(cultivar)Erynginum planum "Blauer Zwerg":insect_groupWild bees == 0   0.002
## as.factor(cultivar)Salvia yangii:insect_groupWild bees == 0                    -2.682
##                                                                               Pr(>|z|)
## (Intercept) == 0                                                                0.9980
## as.factor(cultivar)Aster ageratoides == 0                                       0.9980
## as.factor(cultivar)Campanula persicifolia == 0                                  0.3831
## as.factor(cultivar)Echinacea purpurea "Alba" == 0                               0.2694
## as.factor(cultivar)Erynginum planum "Blauer Zwerg" == 0                         0.9980
## as.factor(cultivar)Salvia yangii == 0                                           0.0439
## insect_groupWild bees == 0                                                      0.0902
## as.factor(cultivar)Aster ageratoides:insect_groupWild bees == 0                 0.0902
## as.factor(cultivar)Campanula persicifolia:insect_groupWild bees == 0            0.3964
## as.factor(cultivar)Echinacea purpurea "Alba":insect_groupWild bees == 0         0.2694
## as.factor(cultivar)Erynginum planum "Blauer Zwerg":insect_groupWild bees == 0   0.9980
## as.factor(cultivar)Salvia yangii:insect_groupWild bees == 0                     0.0439
##                                                                                
## (Intercept) == 0                                                               
## as.factor(cultivar)Aster ageratoides == 0                                      
## as.factor(cultivar)Campanula persicifolia == 0                                 
## as.factor(cultivar)Echinacea purpurea "Alba" == 0                              
## as.factor(cultivar)Erynginum planum "Blauer Zwerg" == 0                        
## as.factor(cultivar)Salvia yangii == 0                                         *
## insect_groupWild bees == 0                                                    .
## as.factor(cultivar)Aster ageratoides:insect_groupWild bees == 0               .
## as.factor(cultivar)Campanula persicifolia:insect_groupWild bees == 0           
## as.factor(cultivar)Echinacea purpurea "Alba":insect_groupWild bees == 0        
## as.factor(cultivar)Erynginum planum "Blauer Zwerg":insect_groupWild bees == 0  
## as.factor(cultivar)Salvia yangii:insect_groupWild bees == 0                   *
## ---
## Signif. codes:  0 '***' 0.001 '**' 0.01 '*' 0.05 '.' 0.1 ' ' 1
## (Adjusted p values reported -- BH method)
```

```
#pairwise
meanie <- emmeans(m2beestop6, pairwise ~   insect_group|cultivar) 
summary (meanie)
```

```
## $emmeans
## cultivar = Anemone japonica:
##  insect_group   emmean       SE  df asymp.LCL asymp.UCL
##  Honey bees    -0.0927    0.292 Inf -6.65e-01     0.480
##  Wild bees     -0.8788    0.371 Inf -1.61e+00    -0.152
## 
## cultivar = Aster ageratoides:
##  insect_group   emmean       SE  df asymp.LCL asymp.UCL
##  Honey bees    -0.0842    0.551 Inf -1.16e+00     0.995
##  Wild bees      0.5184    0.462 Inf -3.87e-01     1.424
## 
## cultivar = Campanula persicifolia:
##  insect_group   emmean       SE  df asymp.LCL asymp.UCL
##  Honey bees    -1.3566    1.045 Inf -3.40e+00     0.692
##  Wild bees     -0.6912    0.760 Inf -2.18e+00     0.799
## 
## cultivar = Echinacea purpurea "Alba":
##  insect_group   emmean       SE  df asymp.LCL asymp.UCL
##  Honey bees     0.4389    0.342 Inf -2.32e-01     1.109
##  Wild bees     -1.6128    0.758 Inf -3.10e+00    -0.128
## 
## cultivar = Erynginum planum "Blauer Zwerg":
##  insect_group   emmean       SE  df asymp.LCL asymp.UCL
##  Honey bees   -19.6665 8003.425 Inf -1.57e+04 15666.758
##  Wild bees     -0.5284    0.699 Inf -1.90e+00     0.841
## 
## cultivar = Salvia yangii:
##  insect_group   emmean       SE  df asymp.LCL asymp.UCL
##  Honey bees     0.8486    0.269 Inf  3.22e-01     1.375
##  Wild bees     -2.8349    1.031 Inf -4.86e+00    -0.813
## 
## Results are given on the log (not the response) scale. 
## Confidence level used: 0.95 
## 
## $contrasts
## cultivar = Anemone japonica:
##  contrast               estimate       SE  df z.ratio p.value
##  Honey bees - Wild bees    0.786    0.362 Inf   2.169  0.0301
## 
## cultivar = Aster ageratoides:
##  contrast               estimate       SE  df z.ratio p.value
##  Honey bees - Wild bees   -0.603    0.499 Inf  -1.207  0.2276
## 
## cultivar = Campanula persicifolia:
##  contrast               estimate       SE  df z.ratio p.value
##  Honey bees - Wild bees   -0.665    1.248 Inf  -0.533  0.5940
## 
## cultivar = Echinacea purpurea "Alba":
##  contrast               estimate       SE  df z.ratio p.value
##  Honey bees - Wild bees    2.052    0.766 Inf   2.679  0.0074
## 
## cultivar = Erynginum planum "Blauer Zwerg":
##  contrast               estimate       SE  df z.ratio p.value
##  Honey bees - Wild bees  -19.138 8003.425 Inf  -0.002  0.9981
## 
## cultivar = Salvia yangii:
##  contrast               estimate       SE  df z.ratio p.value
##  Honey bees - Wild bees    3.684    1.020 Inf   3.612  0.0003
## 
## Results are given on the log (not the response) scale.
```
